# Ozone Aeration Enhance Flowability, Structure, and Antioxidant Activity in Blueberry Pulp Powder

**DOI:** 10.3390/foods14081419

**Published:** 2025-04-20

**Authors:** Newton C. Santos, Raphael L. J. Almeida, Anna E. S. Tomé, Fábio G. Teles, Railene H. C. R. Araújo, Juanne Q. Farias, Maria T. S. d. Fonseca, Virgínia M. d. A. Silva, Victor H. d. A. Ribeiro, Márcia R. d. S. Pedrini, Josivanda P. Gomes, Ana P. T. Rocha

**Affiliations:** 1 Laboratório de Processamento de Biomassa, Universidade Federal de Campina Grande, Campina Grande 58429-900, PB, Brazil; annaemanuelle25@gmail.com (A.E.S.T.); mtfonseca18@gmail.com (M.T.S.d.F.); ana_trindade@yahoo.com.br (A.P.T.R.); 2 Laboratório de Engenharia Bioquímica, Universidade Federal do Rio Grande do Norte, Natal 59078-970, RN, Brazil; raphaelqindustrial@gmail.com; 3 Laboratório de Processamento e Armazenamento de Produtos Agrícolas, Universidade Federal de Campina Grande, Campina Grande 58429-900, PB, Brazil; juanne-queiroz@hotmail.com (J.Q.F.); josivanda@gmail.com (J.P.G.); 4 Laboratório de Qualidade e Processamento de Alimentos de Origem Vegetal, Universidade Federal de Campina Grande, Campina Grande 58429-900, PB, Brazil; ftpesca2019@gmail.com (F.G.T.); railene.herica@professor.ufcg.edu.br (R.H.C.R.A.); 5 Faculdade de Engenharia de Alimentos, Universidade Estadual de Campinas, Campinas 13083-872, SP, Brazil; virginia.mirtes2015@gmail.com; 6 Centro de Ciências Humanas, Sociais e Agrárias, Universidade Federal da Paraíba, Bananeiras 58051-900, PB, Brazil; victor_herbert@hotmail.com; 7 Laboratório de Bioprocessos, Universidade Federal do Rio Grande do Norte, Natal 59078-970, RN, Brazil; marcia.pedrini@ufrn.br

**Keywords:** green technology, drying agents, rice starch, rice protein, fruit pulp powder

## Abstract

Spray drying (SD) is widely used for fruit powder production, but hygroscopic compounds can affect flowability and cause stickiness. This study evaluated rice protein and rice starch as encapsulating agents during SD of blueberry pulp (BPP and BPS, respectively), combined with ozone aeration (BPP-O3 and BPS-O3), focusing on physical, morphological, structural, and bioactive properties, as well as 56-day stability. The process yield was 55.26% (BPP) and 52.5% (BPS) (*p* < 0.05). All microparticles had low moisture (<5.03%) and water activity (<0.21%). BPP had higher phenolic (308.60 mg GAE/100 g) and anthocyanin content (85.26 mg/100 g), while BPS had more flavonoids (33.84 mg CE/100 g). Ozone treatment increased solubility (89.10–91.27%) and reduced hygroscopicity (9.25–10.06%). Morphological analysis revealed that BPP produced smaller, uniform particles (11.70 µm), whereas BPS generated larger (16.67 µm) and more agglomerated particles. Ozone improved sphericity, reduced agglomeration, and enhanced flow properties. FT-IR analysis indicated no new functional groups but a reduction in absorbance bands. Ozone also enhanced the stability of bioactive compounds, reducing anthocyanin and flavonoid degradation over 56 days. Overall, BPP-O3 is a promising approach for producing functional powders with enhanced stability and physical properties, suitable for food applications.

## 1. Introduction

Blueberries (*Vaccinium* spp.) are widely recognized for their high nutritional value and characteristic flavor. They are particularly rich in polyphenols, especially anthocyanins, which play a key role in their potent antioxidant activity [1]. Anthocyanins account for a significant fraction of the fruit’s total phenolic content, often exceeding 50%, and are responsible for its intense purple coloration. Additionally, these compounds are among the primary contributors to the fruit’s bioactivities, which include antioxidant, antiproliferative, and antidiabetic effects [1,2]. Other phenolic compounds, such as phenolic acids, tannins, and non-anthocyanin flavonoids, are also present in blueberries and contribute to their biological properties [3].

However, their high perishability and sensitivity to environmental conditions, such as temperature and humidity, limit their availability and complicate storage and transportation [4]. An efficient strategy to overcome these challenges is the conversion of blueberry pulp into powder, which extends its shelf life and facilitates its incorporation into food and nutraceutical formulations [5]. Food powders derived from fruit pulps can be obtained through various drying methods, including freeze-drying, fluidized bed drying, and spray drying [6]. Among these techniques, SD stands out as one of the most efficient methods for powder production, offering advantages such as high scalability, lower operational costs, and good preservation of bioactive compounds [7]. The process involves atomizing the liquid into fine droplets, which are rapidly dehydrated by a hot air stream, resulting in dry particles with specific characteristics. Moreover, unlike freeze-drying, which requires high energy consumption, SD enables continuous and cost-effective processing, making it a more viable alternative for the industry [8].

Despite its advantages, SD presents challenges related to the physical properties of the final powder, such as limited flowability, stickiness, and lump formation. These issues arise due to the hygroscopic nature of certain compounds and particle adhesion, which can compromise the efficiency of powder transportation, storage, and reconstitution [9]. The selection of appropriate drying agents, such as rice protein and rice starch, can help improve material stability, but does not eliminate these limitations [10]. To mitigate these effects, various aeration techniques have been explored to modify powder structure and enhance its flow properties [9].

Conventional aeration methods include nitrogen discharge and the use of inert gases to reduce particle cohesiveness and minimize lump formation. However, these methods have limitations, such as high operational costs and the need for specialized equipment [11]. Although emerging studies have reported improvements in powder flowability through physical modifications, there is a noticeable lack of research investigating alternative, cost-effective, and sustainable aeration technologies that can be easily integrated into food processing lines. In this context, ozone emerges as a promising yet underexplored alternative for the aeration of food powders [9].

Ozone is a green and emerging technology recognized for its ability to intensify processes and modify the physicochemical characteristics of materials without the need for chemical additives. Its application can reduce particle stickiness, improve flowability, and homogenize the particle size distribution of the powder [12]. Additionally, ozone exhibits antimicrobial potential, which can contribute to the microbiological stability of the final product [13]. While ozone has been extensively studied for surface decontamination and shelf-life extension in fresh produce, few studies have investigated its impact on the structural and functional properties of powdered food matrices, particularly those obtained via spray drying. This gap highlights the need for further research into the use of ozone as a post-drying treatment to improve the technological functionality of fruit-derived powders.

Therefore, the present study aims to address this gap by evaluating the impact of ozone aeration on spray-dried blueberry pulp powders, using rice protein and rice starch as drying agents. The central hypothesis of this study is that ozone application will induce structural modifications in the powder, reducing its cohesiveness and improving its flow properties without compromising its antioxidant characteristics. Additionally, ozone is expected to promote a more homogeneous particle distribution, minimizing lump formation and enhancing material processability. By exploring the feasibility of this novel application, this study seeks to contribute to the development of more functional, stable, and sustainable powdered ingredients for the food and nutraceutical industries.

## 2. Materials and Methods

### 2.1. Materials

Fully ripened and frozen Briteblue blueberries (*Vaccinium* spp.) were purchased from DeMarchi (São Paulo, Brazil). Rice protein (80% protein) was provided by Laboratório Schraibmann (Scharaiber, Cotia, São Paulo, Brazil). Enzymatically modified red rice starch was supplied by [14]. All chemicals, organic solvents, and materials were obtained from Sigma-Aldrich (St. Louis, MO, USA), and the reagents were freshly prepared on the day of analysis.

### 2.2. Blueberry Pulp Preparation

Frozen blueberries, totaling approximately 10 kg, were thawed at room temperature, washed, and sanitized by immersion in a 200 ppm sodium hypochlorite solution for 15 min. The fruits were then pulped using a fruit pulper (model MF-450-F, Tortugan, São Paulo, Brazil). The obtained pulp was pasteurized at 75 °C for 20 s and stored in sterilized Schott bottles. Samples were kept at −18 °C until further use.

### 2.3. Pulp Drying Process

For the drying process, the blueberry pulp was thawed at 4 °C for 12 h and filtered up to four times through an ultrafine nylon mesh (pore diameter of 150 μm) to retain insoluble solids and reduce nozzle clogging. For each experiment, 300 mL of filtered pulp and the corresponding proportion of rice protein (RP) or rice starch (RS) (8% by mass) were homogenized using an Ultra-Turrax T-25 (IKA, Königswinter, Germany) at 8000 rpm for 5 min. This concentration was determined based on the total soluble solids content (11° Brix) and preliminary tests.This resulted in two experimental treatments: BPP (blueberry pulp with rice protein) and BPS (blueberry pulp with rice starch).

The SD was conducted using a spray dryer ADL311S-A (Yamato Scientific^®^, Tokyo, Japan) with a co-current air flow and a two-fluid nozzle to obtain the powders. The internal diameter of the nozzle was 1 mm. The drying conditions were defined based on preliminary tests and previous studies by [15]. Based on the results, an inlet temperature of 180 °C, a feed flow rate of 0.25 L/h, and a drying air flow rate of 3.00 m^3^/min (pressure of 0.3 MPa) were used. The outlet temperature was 70 °C. The feed was continuously stirred on a magnetic stirrer (model C-MAG HS 7, IKA, São Paulo, Brazil). The obtained powders were collected under steady-state conditions, ensuring that the dryer’s operational parameters were stabilized, and that the product was consistently formed. The product was stored in polyethylene terephthalate/aluminum foil/polyethylene laminated bags at 4 °C. Drying experiments were performed in triplicate.

### 2.4. Ozone Dosage Procedure

The final dry powders obtained by SD were subjected to ozone treatment following the optimized experimental protocol proposed by [9]. An ozone generator (Barradent, Shanghai, China) with alternating current was used, pre-calibrated with a continuous rate of 24 g ozone/h. In triplicate, 150 g of microparticles were placed in a cylindrical PVC chamber (400 × 500 mm, diameter × height), equipped with an upper inlet for the ozone generator hose. Throughout the process, the ozone concentration was maintained at 5 g ozone/h, the temperature at 25 °C, and the treatment time at 20 min. After the treatment, the powders were stored in polyethylene terephthalate/aluminum foil/polyethylene laminated bags at 4 °C and were labeled as follows: BPP-O3 (blueberry pulp with rice protein, subjected to ozone treatment) and BPS-O3 (blueberry pulp with rice starch, subjected to ozone treatment).

### 2.5. Characterization of the Obtained Powders

#### 2.5.1. Moisture Content and Water Activity

Moisture content was determined gravimetrically by drying 1 g of the sample at 105 °C until a constant weight was reached. Water activity was measured using a water activity analyzer (AquaLab, Decagon Devices, Pullman, WA, USA) at room temperature (25 °C). All measurements were performed in triplicate.

#### 2.5.2. Solubility and Hygroscopicity

The solubility of the samples was determined by the gravimetric method [16], and hygroscopicity was determined using a saturated NaCl solution to generate a relative humidity of 75% at room temperature (25 °C) [5]. The experiments were conducted in triplicate.

#### 2.5.3. Bulk and Tapped Density

Bulk and tapped densities were determined following the method described by [17]. For this, a 50 mL graduated cylinder was filled with 10.0 g of powder. The powder volumes were recorded for the level reached after gentle tapping (bulk density) or after manual application of pressure to achieve the maximum volume reduction (tapped density). The results are expressed as weight per volume of powder (g/cm^3^). The experiments were conducted in triplicate.

#### 2.5.4. Hausner Ratio and Carr Index

The Hausner ratio (HR) and Carr index (CI) indicate the flowability and cohesiveness of the powder, and their values were calculated according to Equations (1) and (2), respectively [18]. The experiments were conducted in triplicate.

HR = (Tapped density)/(Bulk density)(1)

CI = (Tapped density-Bulk density)/(Bulk density) × 100(2)

#### 2.5.5. Size of Particles

The size of particles was measured using Mastersizer (Mastersizer 3000, Malvern Instruments, Worcester, UK), a laser diffraction particle size analyzer equipped with the automated wet dispersion unit Hydro EV with 1.45 as the relative refractive index.

### 2.6. Scanning Electron Microscopy (SEM) Analysis

The powder morphology was studied using a scanning electron microscope (VEGA3 TESCAN, Medford, MA, USA). A voltage of 15 kV was applied with a vacuum of 0.009 Pa and a magnification of 1500×. The powder was coated with gold nanoparticles using a Desk V thin film deposition solution (Denton Vacuum, Moorestown, NJ, USA) to enhance the electrical conductivity of its surface.

### 2.7. Fourier Transform Infrared (FT-IR) Spectrometer

The chemical characteristics of the powder were assessed using a Fourier transform infrared (FT-IR) spectrometer, model Spectrum 400 (Perkin Elmer, Tokyo, Japan). In brief, powder samples were mixed with potassium bromide (KBr) and then compressed into a disk. FT-IR spectra were recorded within the range of 600 to 3600 cm^−1^, with a spectral resolution of 4 cm^−1^, 32 scans per spectrum (over 34 s), at 22 °C and 60% relative humidity.

### 2.8. Bioactive Compounds with Antioxidant Potential

#### 2.8.1. Determination of Total Phenolic Content (TPC)

The TPC was determined following the method described by [5]. An aliquot, i.e., 0.5 mL of each extract (water extracts of samples were produced by solubilizing them in distilled water at 10%), was transferred to a test tube containing 10 times the Folin–Ciocalteu reagent and 2 mL (7.5%) of sodium carbonate and left to react for 30 min at 25 °C. After the incubation period, the absorbance of the TPC was measured at 750 nm using a microplate reader (model LX Agilent BioTek, Winooski, VT, USA). For the calibration curve, gallic acid (0.02–0.6 mg/mL) was used as a standard. The results were expressed as mg of gallic acid equivalents (GAE)/100 g (mg GAE/100 g).

#### 2.8.2. Determination of Total Flavonoid Content (TFC)

Flavonoids were estimated by the procedure of [19]. For this, 2 g of each sample was mixed in 5 mL of distilled water. Then, 0.3 mL of 5% sodium nitrate and 0.6 mL of 10% aluminum chloride were poured into the flask and left to stand in a dark place at room temperature. Additionally, 2 mL of sodium hydroxide (1 M) was added, and the final volume was adjusted to 10 mL using deionized water. Then, 1 mL of the reaction mixture was mixed with 9 mL of deionized water, and the absorbance was recorded at 510 nm using a microplate reader (model LX Agilent BioTek, USA) against catechin equivalents (CE) as a standard. The results were expressed in mg CE/100 g.

#### 2.8.3. Determination of Anthocyanins

The determination of anthocyanin content was evaluated using the differential pH method. Briefly, 10 mL of KCl buffer (pH 1) or CH_3_CO_2_Na buffer (pH 4.5) was added to the sample (10 ± 0.1 mg), homogenized by vortexing, and placed on an orbital shaker. After 15 min, the sample was centrifuged for 5 min at 7000 rpm and filtered. Finally, the absorbance was measured at 510 and 700 nm using a UV–Vis microplate reader (model LX Agilent BioTek, USA). A calibration curve of cyanidin-3-O-glucoside was obtained with concentrations ranging from 3.125 to 500 µg/mL; thus, the results were expressed in mg/100 g [20].

#### 2.8.4. 2,2-Diphenyl-1-picrylhydrazyl (DPPH) and 2,2-Azinobis (3-ethylbenzothiazo line-6-sulfonic acid)

The antioxidant potential was evaluated in all samples by 2,2-diphenyl-1-picrylhydrazyl (DPPH) and 2,2-azinobis (3-ethylbenzothiazo line-6-sulfonic acid) (ABTS) methods following [21]. Absorbance values of DPPH and ABTS assays were measured by spectrophotometry (SP-2000 UV, Spectrum, Shanghai, China) at 515 and 734 nm, respectively. The results were expressed as micromole Trolox equivalents (μmol TE/g), and the standard curve was linear between 25 and 800 mM Trolox.

### 2.9. Storage Stability

All samples were stored at 20 °C with a controlled relative humidity of 85% in BOD incubators (Quimis, Q315M18, São Paulo, Brazil) for a period of 56 days. Approximately 1 g of each sample was weighed in Petri dishes (55 mm in diameter) and placed in airtight glass containers containing saturated sodium chloride (NaCl) solutions to maintain the relative humidity at 85%. Regular intervals of 14 days were set for analysis over the 56-day period, during which the levels of total phenolic compounds (TPCs), total flavonoid content (TFC), and anthocyanins were measured. All analyses were performed in triplicate to ensure the reliability of the results.

### 2.10. Statistical Analysis

All experiments were performed in triplicate (n = 3), and the results are expressed as mean ± standard deviation. The results were evaluated by analysis of variance (ANOVA) and subjected to Tukey’s test to determine significant differences (*p* < 0.05) between the samples using Assistat beta 7.7 software (available as freeware from: http://www.assistat.com). Pearson’s correlation analysis was conducted to interpret the changes between phenolic compounds and antioxidant potential, using OriginPro 9 software (OriginLab Corporation, Northampton, MA, USA).

## 3. Results and Discussion

### 3.1. Drying Yield

The process yield is one of the main attributes for determining the efficiency and economic viability of the drying process [22]. In Table 1, it can be observed that the drying yields of the blueberry pulp spray-dried using rice protein and rice starch as encapsulating agents were greater than 50% (*p* < 0.05), indicating a successful process both at the laboratory and pilot scale, as suggested by [23]. Notably, the BPP showed the highest yield (55.26%) (*p* < 0.05), suggesting that rice protein plays a more efficient role in forming a protective matrix during drying. This effect may be associated with its superior emulsifying capacity and greater interaction with dissolved solids, reducing particle adhesion to the equipment walls and minimizing losses in the process [24]. Furthermore, proteins form more cohesive structural networks around the particles, promoting more efficient encapsulation and thermal stabilization during SD [25].

On the other hand, although the yield of BPS was lower than that of BPP, it still showed values above 50%, demonstrating its feasibility as an encapsulating agent. Starch, due to its ability to form gels and interact with the water present in the pulp, may contribute to the retention of encapsulated compounds, reducing losses during drying. However, its lower efficiency compared to rice protein may be related to a lower emulsifying capacity and a less effective formation of a protective barrier against particle adhesion to the atomizer walls [26]. Similar values to those in the present study were reported by [27] for SD of blueberry extract using chickpea protein isolate and pea protein concentration (48.33–53.46%). Therefore, the choice of encapsulating agent directly impacts the drying process yield, and rice protein demonstrated greater efficiency in preserving the encapsulated material, which can be advantageous for industrial applications aiming for higher productivity and reduced losses.

### 3.2. Physical Properties

The evaluation of the physical properties of powders is essential to ensure their quality, stability, and industrial applicability [5]. The physical properties of blueberry pulp powders before and after ozone aeration can be observed in Table 1. All powders exhibited low moisture content, ranging from 4.25% (BPP) to 5.03% (BPS) (*p* < 0.05), and low water activity, below 0.21 (*p* > 0.05). Thus, they met the requirements related to adequate stability and the minimization of microbial and chemical reactions [23]. From our results, it can be observed that, before ozone aeration, BPS exhibited higher moisture content and water activity compared to BPP (*p* < 0.05). This can be attributed to the hygroscopic nature of rice starch, which may retain more water in the particulate matrix compared to rice protein [14].

**Figure 1:**
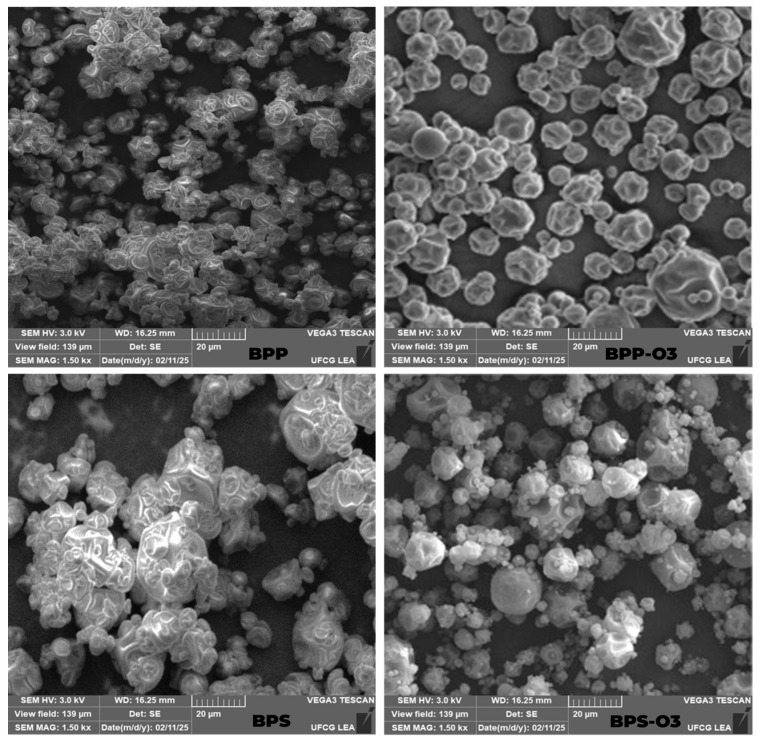
Scanning electron microscopy (SEM) images of blueberry pulp powder obtained by spray drying. BPP: blueberry pulp with rice protein; BPS: blueberry pulp with rice starch; BPP-O3: blueberry pulp with rice protein, subjected to ozonation treatment; BPS-O3: blueberry pulp with rice starch, subjected to ozonation treatment.

These results are consistent with previous studies on the SD of American elderberry juice using soy protein and tapioca starch [28]. After ozone treatment, no clear behavior was observed for moisture content (4.40% (BPP-O3) and 4.97% (BPS-O3), *p* < 0.05). This effect may be due to the oxidizing action of ozone, which can disrupt interparticle interactions and alter the structure of biopolymers, leading to a more open and porous matrix [12]. However, the water activity remained statistically similar between the samples (*p* > 0.05), suggesting that ozone did not significantly alter the availability of free water. This behavior is corroborated by the SEM images (Figure 1), which revealed morphological changes in the particles after ozone treatment. The increased porosity and the presence of surface cavities, especially in BPP-O3, may influence moisture retention and diffusion dynamics within the powder matrix.

A significant increase in solubility was observed in both formulations after ozone treatment (Table 1), with values rising from 76.17% to 89.10% for BPP and from 74.19% to 91.27% for BPS (*p* < 0.05). Initially, BPS exhibited slightly lower solubility compared to BPP, which can be attributed to the limited solubility and emulsifying capacity of rice starch at room temperature [29]. In contrast, rice protein—due to its amphiphilic nature—has a greater ability to interact with both hydrophilic and hydrophobic phases, thereby favoring dispersion in aqueous media [30]. Moreover, in the present study, the rice starch was homogenized with blueberry pulp at room temperature, a condition under which starch tends to have restricted solubilization, further explaining the lower solubility of BPS prior to ozone treatment.

Following ozone exposure, the observed increase in solubility was likely due to oxidative modifications in the structure of the carrier agents and the matrix. Ozone, as a strong oxidizing agent, can promote partial depolymerization of polysaccharides [31] and oxidation of functional groups, introducing polar sites that increase the hydrophilicity of the powders [9]. These structural alterations enhance the interaction between the powder and water, improving its dispersibility and dissolution rate. This hypothesis is supported by scanning electron microscopy (SEM) images (Figure 1), which revealed a more porous and disrupted surface morphology in the ozonized particles (BPP-O3 and BPS-O3), particularly in BPP-O3. The increased porosity likely facilitated water penetration into the matrix, further contributing to the improved solubility of the powders.

While the ozone treatment increased solubility, our findings indicate that it also significantly reduced the hygroscopicity of the powders (*p* < 0.05). Before treatment, the hygroscopicity values were 14.60% (BPP) and 13.80% (BPS), decreasing to 10.06% (BPP-O3) and 9.25% (BPS-O3) after exposure to ozone (*p* < 0.05). The higher initial hygroscopicity of the BPP powders can be attributed to the hydrophilic nature of the proteins, which have polar groups capable of interacting with ambient moisture [30]. On the other hand, rice starch, although a polysaccharide, has a lower tendency to absorb water due to its semi-crystalline structure, resulting in lower hygroscopicity for the BPS powders.

Table 1 shows that the hygroscopicity of the powders ranged from 9.25% (BPS-O3) to 14.60% (BPP) (*p* < 0.05). Notably, the powders containing rice starch exhibited significantly lower hygroscopicity (*p* < 0.05) than those containing rice protein. The lower hygroscopicity observed in the powders with rice starch can be attributed to the semi-crystalline structure of the starch, which limits water absorption [29]. In contrast, proteins such as rice protein have hydrophilic functional groups that facilitate interaction with water molecules, resulting in higher hygroscopicity [32]. Silva et al. [33] reported hygroscopicity values ranging from 7.77% to 13.64% for concentrated rosemary leaf extract and dried muscadine grape pomace powder using pea protein and insect protein as encapsulating agents.

After ozone treatment, the reduction in hygroscopicity observed in both formulations can be attributed to oxidative reactions occurring on the surface of the powder particles. Ozone may induce the formation of covalent cross-links between functional groups of proteins and/or polysaccharides, especially through the oxidation of amino and hydroxyl groups. This cross-linking process reduces the number of free polar sites available for water binding, leading to decreased water sorption capacity and, consequently, lower hygroscopicity [34]. Moreover, ozone can modify the surface topography of the particles, promoting a smoother and more compact structure, which acts as an additional barrier to moisture absorption. These effects are supported by SEM images (Figure 1), which show fewer surface irregularities and less porosity in the ozonized samples compared to the untreated ones.

The bulk density and tapped density of the blueberry pulp microparticles varied according to the encapsulating agent and ozone treatment (Table 1). The BPS samples exhibited a slightly lower bulk density (0.270 g cm^−3^) than the BPP samples (0.275 g cm^−3^), although no statistically significant difference was observed (*p* > 0.05). After ozone treatment, a significant increase in the bulk density was observed in both samples (*p* < 0.05), reaching 0.300 g cm^−3^ (BPP-O3) and 0.304 g cm^−3^ (BPS-O3). This increase can be justified by structural modifications promoted by ozone. Oxidation can alter the surface charge of the particles, reducing electrostatic repulsions and favoring attractive interactions, such as hydrogen bonding and van der Waals forces [35]. This facilitates particle aggregation, contributing to greater packing and, consequently, an increase in bulk density.

On the other hand, the tapped density followed an opposite trend, significantly decreasing after ozone treatment, from 0.393 g cm^−3^ (BPP) and 0.389 g cm^−3^ (BPS) to 0.330 g cm^−3^ (BPP-O3) and 0.325 g cm^−3^ (BPS-O3) (*p* < 0.05). This reduction may be related to modifications in the shape and size distribution of the particles after ozonation, facilitating their rearrangement under compression [23]. This behavior was also observed by [9] when evaluating ozone treatment in spray-dried sugarcane juice powders using maltodextrin and carrot fibers. Overall, our results suggest that ozone treatment can help improve the flow and storage properties of powders, making them more soluble, less hygroscopic, and more stable, factors essential for the industrial application of encapsulated powders.

### 3.3. Flowability and Cohesiveness: Hausner Ratio and Carr Compressibility Index

The flow parameters of blueberry pulp microparticles, evaluated by the Hausner ratio (HR) and Carr compressibility index (CI), indicate significant differences (*p* < 0.05) between the rice-protein- and rice-starch-encapsulated samples, as well as the impact of ozone treatment (Table 1). Before ozonation, the BPP and BPS samples had HR values of 1.43 and 1.44, respectively, and CI values of 42.90% and 44.07%, indicating a poor flow behavior (HR > 1.4 and CI > 35%). This suggests that the powders initially had high cohesiveness and a tendency to compact, which may be attributed to the possible presence of surface charges that favor attractive interactions between the particles. After ozone treatment, a significant improvement in flow properties was observed, with HR reduced to 1.10 (BPP-O3) and 1.06 (BPS-O3), and CI reduced to 10.00% (BPP-O3) and 6.91% (BPS-O3). These values indicate that the ozonized powders exhibited very good flow properties (HR < 1.2 and CI < 15%), as classified in the literature by [36].

The improvement in the flowability of the powders after ozonation can be explained by structural modifications in the particles induced by ozone. Ozone is an oxidizing agent capable of altering the surface composition of encapsulating biopolymers, reducing particle adhesion and minimizing interparticle forces, such as hydrogen bonds and electrostatic interactions [35]. Furthermore, the reduction in the compacted density of the ozonized samples may indicate a lower tendency for excessive packing, contributing to more efficient flow.

The results demonstrate that ozonation was effective in modifying the physical properties of blueberry pulp powders, significantly improving their flowability and reducing their cohesiveness. This improvement could have important practical applications, particularly in industrial processes that require powders with good flow capacity, such as samples for powdered foods and nutritional supplements.

### 3.4. Size Particle and SEM Analysis

The particle size analysis (Table 1) and morphology by SEM (Figure 1) revealed that both the encapsulating agent and the ozone treatment significantly influenced these characteristics. Initially, the BPP particles showed a smaller average size (11.70 µm) and irregular morphology, with less spherical particles and some evidence of aggregation. This suggests that rice protein, acting as the encapsulating agent, was well distributed during the atomization process, resulting in more uniform particles but with a less defined structure, making them susceptible to adhesion. According to [37], the addition of rice protein improves thermal stability and process efficiency but may not completely prevent the formation of aggregates due to interparticle forces.

In contrast, the BPS particles exhibited a significantly larger size (16.67 µm) (*p* > 0.05), indicating a higher tendency for agglomeration. This behavior can be attributed to the nature of starch, whose granules have lower solubility compared to protein, resulting in particles that adhere to each other due to capillary interactions and cohesive forces. Additionally, agglomeration may be associated with the heterogeneous distribution of material during the drying process, compromising the uniformity of the particle morphology [26].

After the ozone treatment, significant modifications were observed in the particle structure. For the sample containing rice protein (BPP-O3), the treatment induced an increase in the average particle size (14.55 µm), along with a more spherical morphology and a lower incidence of agglomeration. This effect can be attributed to the oxidative action of ozone, which may modify the protein structure, enhancing its solubility and promoting a rearrangement of the particle surface, resulting in a more defined geometry and reduced tendency to adhesion. Furthermore, the presence of cavities on the particle surfaces may indicate a restructuring process of the encapsulating matrix, favoring dispersion and reducing interparticle cohesion [12].

In the samples containing rice starch (BPS-O3), the ozone treatment had the opposite effect compared to the rice protein sample, significantly reducing the particle size to 12.03 µm. This behavior suggests that the oxidation induced by ozone increased the solubility of the starch granules, promoting particle dispersion and reducing agglomeration. This phenomenon is consistent with previous studies, which indicate that ozonization can modify the structure of polysaccharides, altering their solubility and interaction with the dispersing phase, resulting in more homogeneous and less adhesive particles [38,39].

Overall, the results demonstrate that the encapsulating agent directly influences the morphology and agglomeration tendency of the particles, while the ozone treatment can act differently depending on the composition of the encapsulating matrix. In the case of rice protein, ozone favored the formation of larger and more defined particles, whereas for rice starch, the predominant effect was particle dispersion and a reduction in the average size.

### 3.5. FT-IR Spectrometer

**Figure 2:**
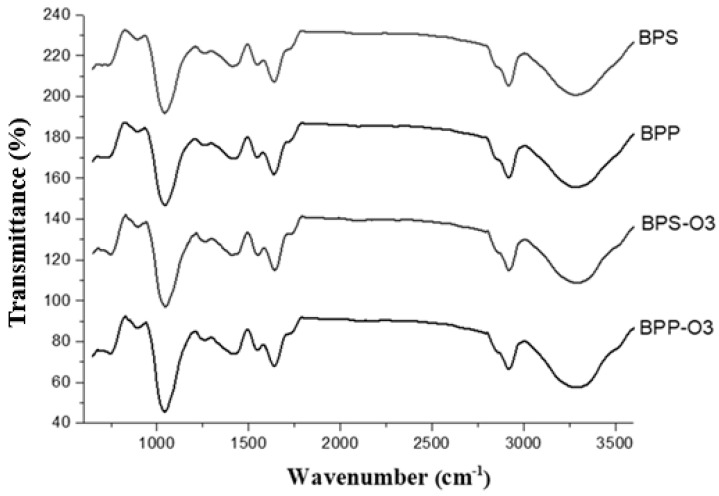
FT-IR spectra of blueberry pulp powders obtained by spray drying and after ozone treatment. BPP: blueberry pulp with rice protein; BPS: blueberry pulp with rice starch; BPP-O3: blueberry pulp with rice protein, subjected to ozonation treatment; BPS-O3: blueberry pulp with rice starch, subjected to ozonation treatment.

FT-IR spectra were used to identify the effects of the encapsulating agent as well as ozonization on the functional groups of the bioactive compounds in the blueberry pulp microparticles. Figure 2 shows that the main bands were identified at 1050 cm^−1^, 1420 cm^−1^, 1645 cm^−1^, 2920 cm^−1^, and 3295 cm^−1^.

The band at 1050 cm^−1^ is attributed to C–O and C–C stretching vibrations, typical of polysaccharides and carbohydrates. The peak at 1420 cm^−1^ is associated with CH_2_ and CH_3_ bending, commonly related to organic acids (e.g., citric and malic acids), as well as pectins and lipids. The band at 1645 cm^−1^ corresponds to the C=O stretching of carboxylate groups (–COO^−^), which are present in carbonyl-containing compounds such as polyphenols and proteins [40,41]. The band at 2920 cm^−1^ is related to C–H stretching in lipid compounds or hydrocarbon chains, while the broad absorption band at 3295 cm^−1^ is influenced by O–H stretching vibrations, indicating the presence of water, phenolic compounds (e.g., anthocyanins), and carbohydrates [42,43]. Cai et al. [44] observed changes at 1447 cm^−1^ due to the stretching (C=C) of an aromatic ring at 1335 cm^−1^, referring to angular deformations of C-O in phenols, and at 1237 cm^−1^, typical of flavonoid compounds after encapsulating blueberry pulp with carboxymethyl starch/xanthan gum.

Comparing the spectra, the addition of rice protein and rice starch caused subtle variations in the intensity of the bands at 1420, 1645, and 3295 cm^−1^, suggesting differences in the interaction of the encapsulating agents with the bioactive compounds, but without the formation of new functional groups. This indicates that the encapsulating agents primarily acted as a protective matrix, preserving the structure of the phenolic compounds [44].

After ozone treatment, a noticeable reduction in band intensity was observed, particularly at 1050, 1645, and 3295 cm^−^^1^. This decrease in absorbance may be associated with the partial degradation or structural modification of bioactive compounds, as ozone is a strong oxidizing agent capable of cleaving chemical bonds, especially in phenolic structures and other oxidation-sensitive compounds [45].

### 3.6. Bioactive Compounds with Antioxidant Potential

Table 2 presents the results for the total phenolic content (TPC), total flavonoid content (TFC), anthocyanins, and antioxidant potential (DPPH and ABTS) of the blueberry pulp microparticles obtained by SD, using rice protein and rice starch as encapsulating agents, before and after ozone treatment.

The BPP sample exhibited significantly higher values of TPC (308.60 mg GAE/100 g), anthocyanins (85.26 mg/100 g), and antioxidant capacity (DPPH and ABTS) compared to BPS (*p* < 0.05). This suggests that rice protein may provide more effective protection against the degradation of bioactive compounds during the drying process due to its film-forming and emulsifying properties [27,28,43]. Proteins can form more cohesive and continuous matrices, which entrap bioactives more efficiently and reduce their direct exposure to heat and oxygen, thereby preserving their integrity during SD [30].

On the other hand, the BPS sample showed a higher total flavonoid content (33.84 mg CE/100 g), suggesting that rice starch better preserves flavonoids during SD. This may be attributed to the semi-crystalline structure of starch and its ability to form protective matrices with lower porosity and higher barrier properties [42], which can limit oxygen diffusion and thermal degradation. Additionally, intermolecular interactions such as hydrogen bonding between starch molecules and flavonoids may further enhance their stabilization by reducing oxidative degradation [40,44].

Previous reports have also indicated that the structural and functional properties of encapsulating agents greatly influence the retention of phenolic compounds, depending on their polarity and sensitivity to oxidative and thermal stresses [43,44]. For example, [46] reported TPC values ranging from 61.29 to 128.97 mg GAE/g in spray-dried acerola juice encapsulated with different protein matrices, reinforcing the role of matrix composition in bioactive retention.

Although the ozone treatment improved the physical and flow properties of the microparticles, it had a negative impact on the bioactive compounds, leading to a significant reduction in TPC, TFC, anthocyanins, and antioxidant capacity values (*p* < 0.05). This effect was more pronounced in the BPS-O3 samples, which exhibited the greatest loss of bioactive compounds compared to the BPP-O3 samples. This may be attributed to the strong oxidative potential of ozone, which can react with phenolic structures and break aromatic rings or hydroxyl groups, leading to molecular degradation and decreased antioxidant capacity [45,47].

Notably, TPC was reduced by approximately 5% in BPP-O3 and 4% in BPS-O3. In the case of flavonoids, the decline was more pronounced in the rice protein microparticles (from 30.17 to 27.13 mg CE/100 g), while those with rice starch maintained values closer to the original, despite a significant reduction (*p* < 0.05). The most significant losses were observed for anthocyanins, with reductions of 9.5% and 8.9% in the rice protein (BPP-O3) and rice starch (BPS-O3) samples, respectively.

The negative impact of ozone was also evident in the antioxidant parameters, with significant reductions in DPPH and ABTS values. The antioxidant capacity by the ABTS method, for example, decreased from 65.38 to 50.47 μmol TE/g (*p* < 0.05) in the rice-protein-encapsulated pulp and from 61.68 to 46.30 μmol TE/g (*p* < 0.05) in the rice-starch-encapsulated pulp. These results indicate that the oxidation promoted by ozone may have degraded some of the phenolic compounds and anthocyanins, reducing the antioxidant capacity of the microparticles [45]. These results indicate that the oxidation promoted by ozone may degrade phenolic compounds and anthocyanins by generating reactive oxygen species (ROS), which accelerate the breakdown of antioxidant molecules and compromise the functional potential of the microparticles [45,47,48].

**Figure 3:**
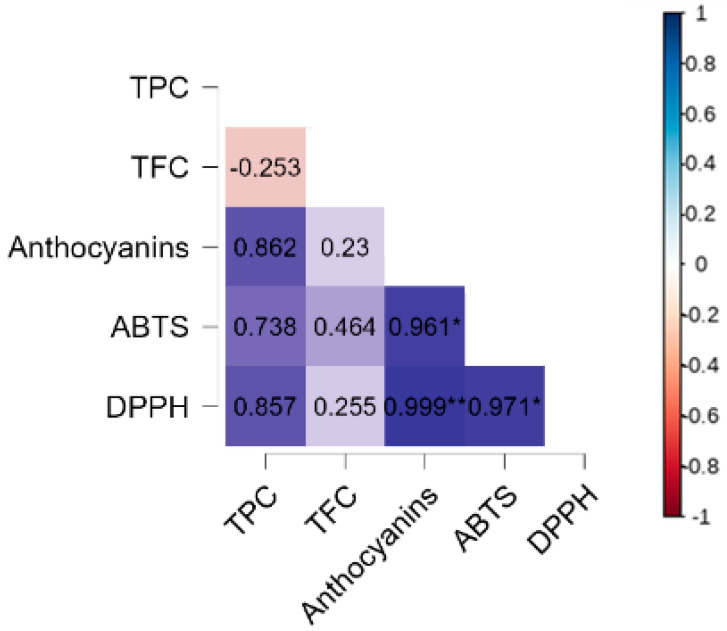
Pearson correlation analysis for bioactive compounds (TPC, TFC, anthocyanins) and antioxidant capacity (ABTS and DPPH). TPC: total phenolic compounds, TFC: total flavonoid content. * *p* < 0.05, ** *p* < 0,01.

A correlation analysis was performed to better understand the relationships between bioactive compounds and antioxidant potential (Figure 3). The results indicated a strong positive correlation between the anthocyanin content and antioxidant capacity, both by the ABTS method (*r* = 0.961, *p* = 0.05) and by DPPH (*r* = 0.999, *p* = 0.001), suggesting that these pigments play a key role in the antioxidant activity of the microparticles. This finding reinforces the importance of anthocyanins as major contributors to the antioxidant capacity of blueberry microparticles, which agrees with previous studies by the authors of [47] that highlight their high reactivity with free radicals.

The TPC showed a positive correlation with anthocyanins (*r* = 0.862), ABTS (*r* = 0.738), and DPPH (*r* = 0.857), although these associations were not statistically significant. This suggests that, despite phenolic compounds contributing to antioxidant activity, the influence of anthocyanins may be more pronounced in this system. On the other hand, TFC did not show a significant correlation with other bioactive compounds or antioxidant capacity. The negative correlation between TPC and TFC (*r* = −0.253) suggests that, in the analyzed microparticles, the presence of one group may not be directly associated with an increase in the other, which could be explained by structural and solubility differences between these compounds.

The correlation results also help us to understand the effects of ozone treatment on the bioactive compounds and antioxidant capacity of microparticles. As previously observed, there was a significant reduction in the levels of TPC, TFC, and anthocyanins after ozonization, which directly impacted the ABTS and DPPH values. However, as anthocyanins showed the highest correlations with antioxidant activity, their degradation due to ozone treatment may have been one of the main factors responsible for the reduction in the antioxidant potential of microparticles. Furthermore, the strong correlation between the ABTS and DPPH values (*r* = 0.971, *p* = 0.05) reinforces that both methods are complementary in evaluating the antioxidant capacity of the samples.

### 3.7. Storage Stability

**Figure 4:**
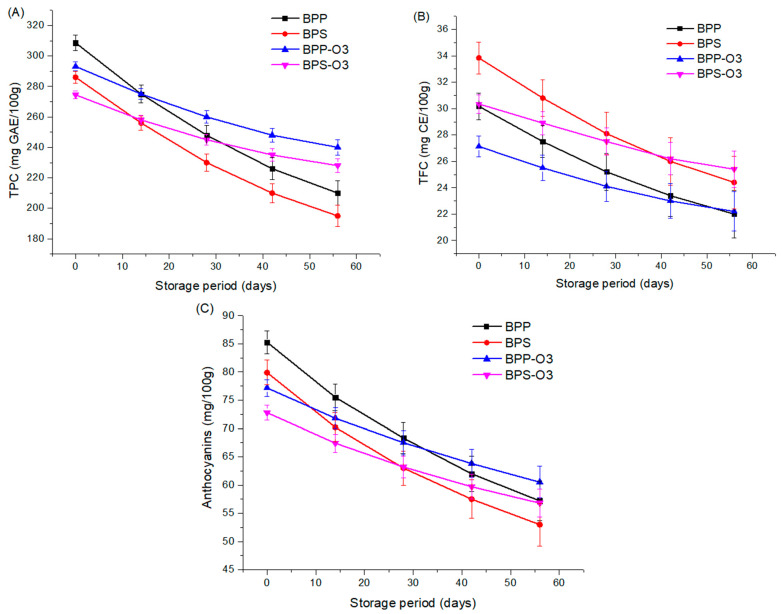
Stability of total phenolic content (TPC) (**A**), total flavonoid content (TFC) (**B**), and anthocyanins (**C**) during controlled storage (56 days) of blueberry pulp powder obtained by spray drying. BPP: blueberry pulp with rice protein; BPS: blueberry pulp with rice starch; BPP-O3: blueberry pulp with rice protein, subjected to ozonation treatment; BPS-O3: blueberry pulp with rice starch, subjected to ozonation treatment.

The stability of bioactive compounds during storage is a crucial factor for ensuring the quality and functionality of powder products [47]. In this study, the stability of TPC (Figure 4A), TFC (Figure 4B), anthocyanins (Figure 4C), and antioxidant activity (DPPH and ABTS) of blueberry pulp microparticles encapsulated with rice protein or rice starch, as well as the microparticles after ozonization treatment, was evaluated during 56-day storage period under controlled conditions (85% relative humidity, 25 ± 2 °C) The choice of a 56-day period was based on previous studies that used similar durations to simulate medium-term storage and assess the degradation kinetics of bioactive compounds in dried fruit powders intended for use in functional foods [47]. This timeframe allows the identification of significant trends in compound stability and is commonly adopted in shelf-life evaluations of encapsulated bioactive products.

The results demonstrated a progressive reduction in bioactive compounds throughout storage, a phenomenon widely reported in the literature due to the oxidative instability and thermal degradation of these metabolites [5]. However, the magnitude of the reduction varied according to the encapsulating agent and the application of ozone treatment. The results indicate (Figure 4A) that the microparticles not treated with ozone underwent greater TPC degradation compared to those subjected to ozone treatment. While the BPP and BPS samples lost approximately 32% of the phenolic compounds, the BPP-O3 and BPS-O3 samples exhibited around 45% less degradation, highlighting a protective effect of ozonization throughout storage.

The choice of encapsulating agent also influenced TPC stability. In general, the samples encapsulated with rice protein (BPP and BPP-O3) demonstrated higher retention of phenolics compared to those containing rice starch (BPS and BPS-O3). At the end of storage, the TPC content of the BPP and BPP-O3 samples was about 7% higher than that of the BPS and BPS-O3 samples, suggesting that rice protein may provide greater protection against oxidative degradation of bioactive compounds. Although ozone treatment initially reduced the phenolic compound content in the samples (Table 2), the storage results show that the ozonized samples exhibited greater stability over time. This effect may be explained by possible structural changes in the encapsulating matrix induced by ozonization [48].

Regarding TFC (Figure 4B), it can be observed that the degradation rate over storage was similar between the two types of encapsulants, indicating that, regardless of the material used, flavonoids are susceptible to oxidative degradation. However, once again, the microparticles subjected to ozone treatment exhibited less degradation over time. The reduction in TFC was about 35% lower in the ozonized microparticles compared to the untreated ones. Furthermore, the BPS-O3 samples showed the highest flavonoid levels at the end of storage, suggesting that the combination of rice starch and ozone treatment may provide greater stability to flavonoids in blueberry pulp microparticles.

Anthocyanins, compounds highly susceptible to degradation [49], exhibited a significant reduction over storage (Figure 4C). After 56 days, the losses were more pronounced in the non-ozonized samples, ranging from 32.9% (BPP) to 33.6% (BPS), while the ozonized microparticles showed a smaller reduction, 21.6% (BPP-O3) and 21.9% (BPS-O3). This result indicates that ozone treatment provided greater stability to anthocyanins during storage, possibly due to the formation of a matrix more resistant to oxidation and other degradative factors. Furthermore, the formation of secondary products from ozonation may have contributed to the stabilization of the compounds, delaying their degradation over time [50].

Previous studies by [51] reported that ozone treatment can enhance the stability and accumulation of bioactive compounds in plant products by improving antioxidant capacity, reducing microbial load, and promoting the production of secondary metabolites related to stress. In summary, the results demonstrate that ozone treatment, although initially reducing bioactive compound levels, provided greater stability during storage, particularly for anthocyanins and TFC. Additionally, the use of rice starch as an encapsulating agent promoted superior retention of bioactive compounds over time.

It is important to highlight a limitation of this study: the storage evaluation was conducted over 56 days at a single condition of temperature and relative humidity. Although this setup simulates ambient storage conditions and aligns with previous shelf-life studies, the absence of multiple storage conditions (e.g., low temperature or varying humidity) may limit the generalization of the findings. Future studies are recommended to explore a broader range of storage conditions and longer durations to better assess the long-term stability and behavior of bioactive compounds in encapsulated fruit powders.

## 4. Conclusions

This study demonstrated that the choice of encapsulating agent and ozone treatment has significant impacts on the physical and chemical properties, as well as the stability of bioactive compounds in spray-dried blueberry pulp microparticles. Rice protein proved to be superior to rice starch in terms of drying yield and the protection of phenolic compounds and anthocyanins. On the other hand, rice starch excelled in preserving flavonoids. Ozonation induced structural modifications in the particles, increasing solubility, reducing hygroscopicity, and improving flow properties, which are highly desirable for industrial applications. The ozone treatment significantly altered particle morphology, enhancing sphericity and reducing agglomeration, particularly in the rice protein sample. These modifications further contributed to increased solubility, reduced hygroscopicity, and improved flow properties, making the powders more suitable for industrial applications.

FT-IR analysis indicated that ozone treatment led to a reduction in absorbance band intensity, resulting from the partial degradation of phenolic compounds and anthocyanins due to oxidative action. Despite initially reducing the levels of bioactive compounds, ozone treatment proved to be an effective strategy for improving their stability during storage. Additionally, ozone conferred greater stability to anthocyanins and flavonoids over time, possibly due to the formation of a matrix more resistant to oxidation. In summary, the combination of rice protein as an encapsulating agent and ozone treatment emerges as a promising approach for industrial applications, particularly in products requiring high solubility, low hygroscopicity, and bioactive compound preservation. Future research may explore the application of these powders in food formulations and supplements, assessing their functionality and consumer acceptance.

## Figures and Tables

**Table 1:** Drying yield, physical properties, flow properties, and particle size of blueberry pulp powder obtained by spray drying using rice protein and rice starch as encapsulating agents, and their respective powders after the ozonation dosing procedure.

Parameters	BPP	BPS	BPP-O3	BPS-O3
Drying yield (%)	55.26 ± 0.27 ^a^	52.50 ± 0.42 ^b^	-	-
Moisture content (%)	4.25 ± 0.06 ^b^	5.03 ± 0.05 ^a^	4.40 ± 0.10 ^b^	4.97 ± 0.04 ^a^
Water activity	0.201 ± 0.01 ^a^	0.208 ± 0.00 ^a^	0.203 ± 0.01 ^a^	0.206 ± 0.02 ^a^
Solubility (%)	76.17 ± 0.37 ^c^	74.19 ± 0.10 ^d^	89.10 ± 0.12 ^b^	91.27 ± 0.21 ^a^
Hygroscopicity (%)	14.60 ± 0.25 ^a^	13.80 ± 0.17 ^b^	10.06 ± 0.38 ^c^	9.25 ± 0.11 ^d^
Bulk density (g cm^−3^)	0.275 ± 0.01 ^b^	0.270 ± 0.01 ^b^	0.300 ± 0.01 ^a^	0.304 ± 0.02 ^a^
Tapped density (g cm^−3^)	0.393 ± 0.01 ^a^	0.389 ± 0.01 ^a^	0.330 ± 0.02 ^b^	0.325 ± 0.01 ^b^
Hausner ratio	1.43 ± 0.11 ^a^	1.44 ± 0.19 ^a^	1.10 ± 0.11 ^b^	1.06 ± 0.19 ^b^
Carr index (%)	42.90 ± 0.45 ^a^	44.07 ± 0.63 ^a^	10.00 ± 1.02 ^b^	6.91 ± 0.77 ^c^
Size particle (µm)	11.70 ± 0.14 ^c^	16.67 ± 0.10 ^a^	14.55 ± 0.25 ^b^	12.03 ± 0.33 ^c^

Notes: mean ± standard deviation; BPP: blueberry pulp with rice protein; BPS: blueberry pulp with rice starch; BPP-O3: blueberry pulp with rice protein, subjected to ozonation treatment; BPS-O3: blueberry pulp with rice starch, subjected to ozonation treatment; ^a–d^ different letters in the same row indicate significant differences (*p* ≤ 0.05).

**Table 2:** Total phenolic content (TPC), total flavonoid content (TFC), anthocyanins, and antioxidant potential (DPPH and ABTS) of blueberry pulp powder obtained by spray drying using rice protein and rice starch as encapsulating agents, and their respective powders after ozone treatment.

Parameters	BPP	BPS	BPP-O3	BPS-O3
TPC (mg GAE/100 g)	308.60 ± 5.01 ^a^	286.02 ± 4.20 ^b^	293.11 ± 3.15 ^b^	274.46 ± 2.51 ^c^
TFC (mg CE/100 g)	30.17 ± 1.10 ^b^	33.84 ± 1.20 ^a^	27.13 ± 0.80 ^c^	30.35 ± 0.74 ^b^
Anthocyanins (mg/100 g)	85.26 ± 2.05 ^a^	79.88 ± 2.20 ^b^	77.16 ± 1.50 ^b^	72.78 ± 1.23 ^c^
ABTS (μmol TE/g)	65.38 ± 1.09 ^a^	61.68 ± 0.84 ^b^	50.47 ± 0.30 ^c^	46.30 ± 0.82 ^d^
DPPH (μmol TE/g)	72.85 ± 0.76 ^a^	69.11 ± 1.03 ^b^	64.66 ± 1.50 ^c^	58.71 ± 0.53 ^d^

Notes: mean ± standard deviation; BPP: blueberry pulp with rice protein; BPS: blueberry pulp with rice starch; BPP-O3: Blueberry pulp with rice protein, subjected to ozonation treatment; BPS-O3: blueberry pulp with rice starch, subjected to ozonation treatment. DPPH: 2,2-diphenyl-1-picrylhydrazyl; ABTS: 2,2-azinobis (3-ethylbenzothiazoline-6-sulfonic acid). ^a–d^ different letters in the same row indicate significant differences (*p* ≤ 0.05).

## Data Availability

The original contributions presented in the study are included in the article; further inquiries can be directed to the corresponding author.
